# Construction of lncRNA- and circRNA-associated ceRNA networks in the prostatic urethra of rats after simulating transurethral laser prostatectomy (TULP)

**DOI:** 10.1007/s11010-023-04804-1

**Published:** 2023-07-06

**Authors:** XiaoHu Tang, ZhiYan Liu, Hao Liu, Heng Zhang, Ye Tian, ShuJie Xia, ZhaoLin Sun, GuangHeng Luo

**Affiliations:** 1 Medical College, Guizhou University, Guiyang, 550025 Guizhou Province China; 2 Department of Urology Surgery, Guizhou Province People’s Hospital, Guiyang, 550002 Guizhou Province China; 3 School of Clinical Medicine, Guizhou Medical University, Guiyang, 550025 Guizhou Province China; 4 Department of Urology Surgery, Shanghai First People’s Hospital, Shanghai Jiao Tong University, Shanghai, 201620 China

**Keywords:** BPH, ceRNA, lncRNA, circRNA, Microarray, TULP

## Abstract

Non-coding RNA appears to be involved in wound repair. Competing endogenous RNA (ceRNA) appears to be an important post-transcriptional mechanism, it means that long noncoding RNA (lncRNA) or circular RNA (circRNA) acts as a microRNA (miRNA) sponge to further regulate mRNA. However, ceRNA network related to wound repair after prostatectomy has yet been constructed. TULP is the main surgical method of prostatectomy, but there have been no reports of TULP rat models in the past. We simulated TULP on rats, and observed the whole process of wound injury and repair after operation through pathological examination of wound tissue. Next, we discovered 732 differentially expressed lncRNAs (DElncRNAs), 47 differentially expressed circRNAs (DEcircRNAs), 17 differentially expressed miRNAs (DEmiRNAs), and 1892 differentially expressed mRNAs (DEmRNAs) related to wound repair after TULP through full transcriptome microarray and bioinformatics methods, and confirmed the reliability of transcriptome data by quantitative Reverse Transcription PCR (qRT-PCR), and immunohistochemistry. Then, we constructed the lncRNA- and circRNA-associated ceRNA regulatory networks related to wound repair after TULP in rats. Gene Ontology (GO) and Kyoto Encyclopedia of Genes and Genomes (KEGG) pathway enrichment analyses showed that molecules in these networks were mainly involved in inflammatory infiltration, cell differentiation, and intercellular interactions and involved signal pathways such as the PI3K-Akt signaling pathway. Thus, this study successfully established the TULP model in rats, revealed potentially important biomarkers and ceRNA networks after prostatectomy in rats, and provided theoretical support for the repair of post-prostatectomy wound.

**Supplementary information:**

The online version contains supplementary material available at 10.1007/s11010-023-04804-1.

## Introduction

Benign prostatic hyperplasia (BPH) is a common disease in older men [1], and its incidence increases with age. Approximately 50% of men > 50 years of age have pathological evidence of BPH, increasing to 80% by age 80 [2]. BPH is associated with lower urinary tract symptoms (LUTS), such as dysuria, frequent urination, and urgency [3]. Progression of the disease may lead to urinary tract infections [4], secondary bladder calculi [5], hydronephrosis or renal failure [6], which could adversely affect the patient’s quality of life. Approximately 10% of patients undergo surgery due to disease progression and ineffective medication [7]. Although the gold standard for prostate surgery, transurethral resection of the prostate, has the advantages of less bleeding and higher efficiency [8], the same effect is observed with new technologies such as thulium laser transurethral vaporesection and holmium laser enucleation of the prostate [9, 10]. Some patients still experience urinary tract infection [11], increased urinary frequency [12] and bladder neck contracture [13] after surgery. However, we found that the healing of the post-prostate surgery wound effectively reduced the above complications [14]. We previously found through a dog model of transurethral two‑micron laser resection of the prostate (TmLRP) that the repair process after prostatectomy involves re-epithelialization of the wound, infiltration and regression of inflammation [15]. In short, the repair process is complex. We also found some clinical phenomena affected the repair process after TmLRP using dog model, such as inflammation [16], the molecular mechanisms underlying these phenomena have not been fully elucidated. Thus, the construction of molecular regulatory networks to identify potential biomarkers and regulatory targets is essential to reduce complications and guide clinical treatment.

With the advancement of laser surgical equipment, transurethral laser prostatectomy TULP is widely used in minimally invasive treatment of the prostate. However, there were not many studies of molecular mechanisms on wound repair after prostatectomy, and the research mainly focused on the coding genes. Our previous studies in dog models of TULP indicated that inhibiting reactive oxygen species NF-κB pathway regulates macrophage polarization accelerated prostate urethral wound healing [17], improving oxidative stress levels may delay wound repair after TULP through MAPK signaling [18], urine promote re-epithelialization of prostate wounds may through stimulating the expression of TGF-β1 in prostate stromal cells[19], and the lack of scar tissue after repair of prostate urethral wounds may be related to the high expression of CKIP-1 in a large number of prostate epithelial cells in the wound [20]. However, the vast majority of the molecular mechanisms research underlying wound repair after TULP has focused on the coding genes, and these studies can’t reveal the comprehensive molecular characteristics behind the wonder repair after TULP. Transcriptomic analyses revealed that less than 2% of the molecules involved encoded proteins [21]. Most of the transcripts were noncoding RNAs, including lncRNAs, circRNAs, and miRNAs, which play important roles in the occurrence and development of diseases, and are expected to become biomarkers and treatment targets. For instance, the lncRNA LIPE-AS1 is a potential regulator of adipogenesis [22], plasma-derived exosomal miR-15a-5p is a promising and effective diagnostic biomarker for the early detection of endometrial cancer [23], and circRNA vgll3 promotes osteogenic differentiation of adipose-derived mesenchymal stem cells [24]. Competing endogenous RNAs (ceRNAs) have been demonstrated to be important in molecular regulatory networks. Based on ceRNA theory [25], lncRNAs or circRNAs contain miRNA binding sites, and can act as sponges to adsorb miRNAs and indirectly regulate mRNAs. CeRNAs play an important role in cancer development [26–28] and tissue repair [29–31]. However, the regulatory role of ceRNA mechanisms in the wound repair process after resection of the prostate has not been fully explored.

In this study, we successfully constructed a rat model of TULP, and observed the repair process of prostatic urethral wound in rats. Then, we performed a transcriptome analysis of trauma tissues, and corresponding control tissues using microarray sequencing technology to identify differentially expressed lncRNAs, circRNAs, miRNAs, and mRNAs in the repair. Subsequently, ceRNA networks associated with DElncRNAs or DEcircRNAs were constructed. We used a series of bioinformatics analyses, including protein–protein interaction analysis and functional enrichment analysis. In addition, the microarray results were validated by qRT-PCR, and immunohistochemistry. These studies could provide new insights for theoretical and clinical studies of repair after prostate resection.

## Materials and methods

### Animals and environmental conditions

In total, 23 healthy male Sprague Dawley (SD) rats (10–12 months old, body weight range of 450–480 g) were purchased from Tengxin Biotechnology Co.Ltd (Chongqing, China). They were reared under standard laboratory conditions (12/12 h light/dark cycle, 22 ± 2 °C with a relative humidity of 55 ± 5%), and allowed to consume standard laboratory food pellets and water ad libitum. This research followed the Code of Ethics of the World Medical Association.

### Surgical procedure and tissue collection

**Fig. 1:**
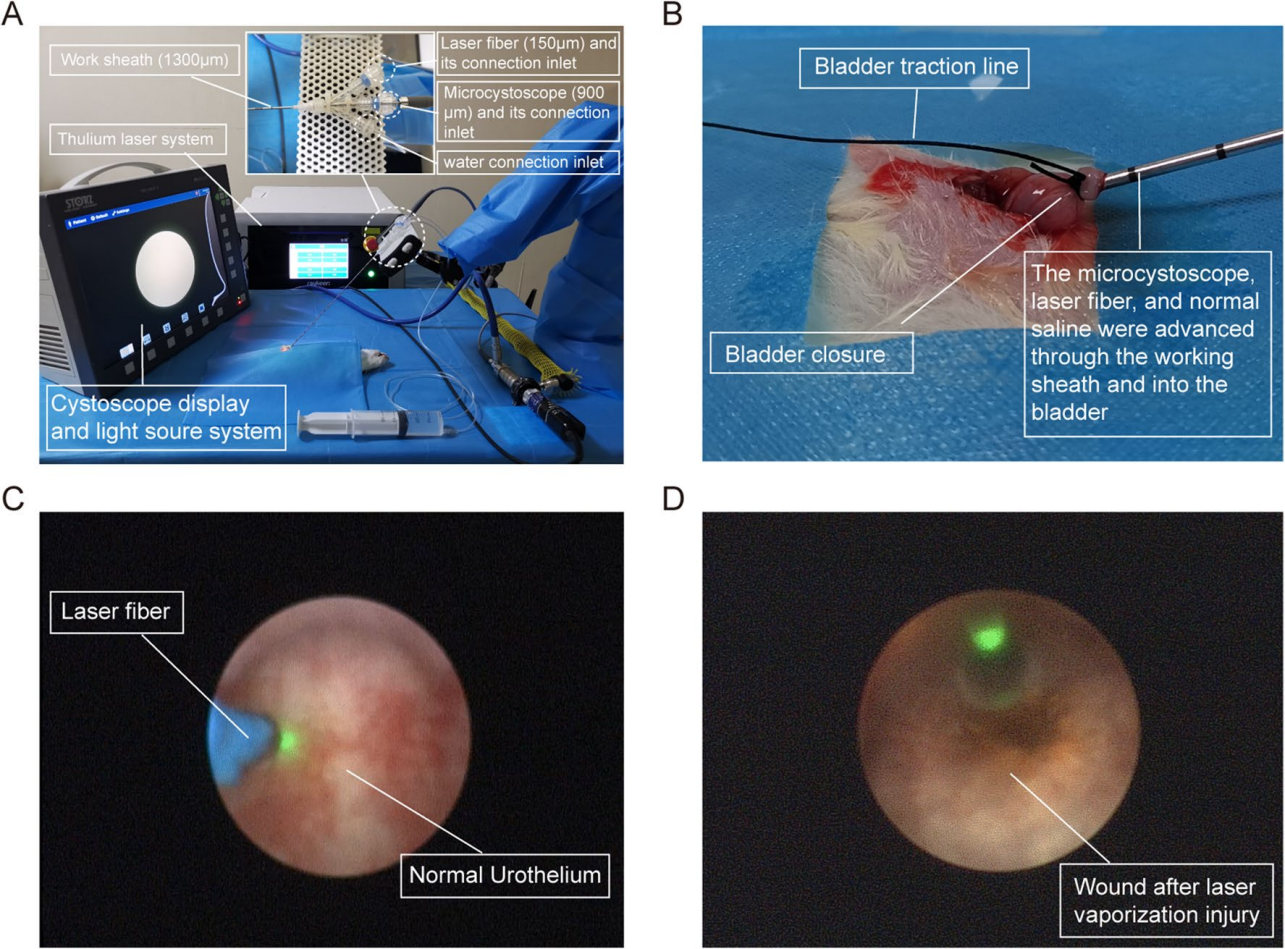
Surgical procedure (The microcystoscope diameter is small, so the image resolution is lower than that of a normal cystoscope). **A** The surgical equipment: microcystoscope system, thulium laser system, and cystoscope system; **B** A lower abdominal incision is made to expose the bladder, and ultramicroendoscope was inserted into bladder and closed the bladder; **C** Observation of the urethra in the prostate direct vision endoscopically; **D** Carbonized layer on the wound surface after the thulium laser vaporized the uroepithelium of the prostate

Fifteen SD rats were randomly selected to establish the rat model of TULP. The rats were fasted from food and water for 6 h before the procedure, and anesthetized with intraperitoneally injected 1% pentobarbital sodium (40 mg/kg). The rats were placed in the supine position, and the limbs were fixed. cystoscope display and light soure system (Karl Storz Endoskope, Tuttlingen, Germany), thulium laser system (1940 nm thulium laser, Raykeen, Shanghai, China), microcystoscope system (PD-D-1083, PolyDiagnost, Berlin, Germany), three-way tube which simultaneously connect syringe, microcystoscope, and laser fiber, and homemade microcystoscope working stent were prepared for the surgery (Fig. 1A). A small incision was made longitudinally in the lower abdomen. After entering the abdominal cavity, the bladder was exposed outside the abdominal cavity. The bladder was incised, and the working sheath was placed inside and ligated for fixation, and suture for ligation of bladder can be used for traction of bladder during operation (Fig. 1B). The bladder was manually irrigated with normal saline under low pressure, and checked for complete bladder closure. The microcystoscope was inserted into the urethra through the bladder neck (Fig. 1C). The thulium laser with a power of 10 W was used to vaporize the uroepithelium of the prostate until the initial carbonized layer appeared on the wound surface (Fig. 1D). After confirming the absence of bleeding on the wound surface, the working sheath was removed, and the bladder and abdominal incision were sutured. Three rats were randomly sacrificed on 1, 3, 5, 7, and 9 days after surgery. The prostate containing the urethra was collected, and fixed in 10% formalin for subsequent pathological examination.

By observing the repair process of urethral wound in rat prostate, 5 days after trauma was the middle stage of wound repair. During this period, new urothelium appeared on the prostate urethral wound, which was the key period of repair. We decided to conduct a follow-up study on the molecular characteristics of the wound at this time point. The other 8 rats were randomly divided into the TULP and sham groups, with 4 rats in each group. The surgical model was established in the TULP group according to the above methods. In the sham group, only an abdominal median incision was performed, without performing the laser vaporization procedure. On the 5 days after the surgery, the wound tissue from the TULP group and the tissue from the same location in the sham group were collected, and placed in liquid nitrogen for subsequent RNA sequencing, thus, experiments were performed four biological replicates for each group.

### Histopathological examination and immunohistochemical staining

The fixed tissue embedded in paraffin was cut into 5-μm-thick sections. After dewaxing and rehydration, sections were stained with hematoxylin and eosin (HE) according to a standard procedure for routine histopathology and observed under Olympus BX53F microscope (Olympus Corporation, Tokyo, Japan).

To determine CK7, UPIII, and ASPM expression, we cut the specimens into 5-μm-thick slices, which were then deparaffinized with xylene and rehydrated by graded ethanol washes. Next, the tissue specimens were incubated with antibodies against CK7 (1:100, ab181598, Abcam company, Washington, USA), UPIII (1:200, ab187299, Abcam company, Washington, USA), and ASPM (1:200, DF10064, Affinity Biosciences, Cincinnati, USA) at 4 °C overnight. Subsequently, the sections were washed with PBS and incubated with HRP-conjugated secondary antibody. The tissues were observed under a light microscope.

### Microarray assay

Total RNA was quantified by a NanoDrop ND-2000 (Thermo Scientific, Massachusetts, USA), and the RNA integrity was assessed using an Agilent Bioanalyzer 2100 (Agilent Technologies, Santa Clara, USA). Sample labeling, microarray hybridization and washing were performed based on the manufacturer's standard protocols. Briefly, total RNA was transcribed to double-stranded cDNA, synthesized into cRNA and labeled with Cyanine-3-CTP. The labeled cRNAs were hybridized onto the microarray. After washing, the arrays were scanned by an Agilent Scanner G2505C (Agilent Technologies, Santa Clara, USA). The Agilent Rat ceRNA Microarray (Design ID: 086243) and miRNA Microarray (Design ID: 070154) were used for this experiment, and the microarray data included lncRNA, circRNA, miRNA, and mRNA. The data analysis of the 8 samples was conducted by OE Biotechnology Co., Ltd. (Shanghai, China). Feature Extraction software (version 10.7.1.1, Agilent Technologies, Santa Clara, USA) was used to analyze array images to obtain raw data, which were normalized with the quantile algorithm.

### Differential expression analysis and hierarchical clustering

Differentially expressed genes were then identified through fold change, and the *p* values were calculated with a *t* test. The threshold set for up- and down-regulated genes was |FoldChange| > 2.0 and a *p* value < 0.05. Volcano plots of differentially expressed RNAs (DERNAs) were constructed by the “ggplot2” package in R software (version 4.1.2, Boston, Massachusetts, USA). Hierarchical clustering analysis was subsequently performed by the “pheatmap” package in R software (version 4.1.2, Boston, Massachusetts, USA).

### Isolation of RNA and qRT-PCR

Total RNA was isolated by TRIzol (Invitrogen) based on the manufacturer’s protocol. CDNA of lncRNA, circRNA, and mRNA were synthesized using a TAKARA reverse-transcriptase-PCR kit (Takara, Japan), while cDNA of miRNA was reversely transcribed miRNA First Strand cDNA Synthesis Kit (Sangon Biotech, China). According to manufacturer's instructions, qRT-PCR was performed using SYBR green (Takara, Japan) from Applied Biosystems. All qRT-PCR data were analyzed by the 2^−△△CT^ method. GAPDH was used as an internal control for data normalization of lncRNA, circRNA, and mRNA, whereas U6 was used as an internal control for miRNA. Primer synthesis was performed by Sangon Biotech. The sequences of the U6 RNA and the universal PCR reverse primer are proprietary information held by Sangon Biotech. The qRT-PCR primer sequences are listed in Table S1. All experiments were performed in triplicate. Results of RT-PCR were analyzed using Graphpad Prism 9.3.0 (Graphpad Prism Inc., San Diego, USA).

### Construction and enrichment analysis of the DElncRNAs-DEmiRNAs-DEmRNAs and DEcircRNAs-DEmiRNAs-DEmRNAs regulatory networks

Based on ceRNA hypothesis, lncRNA (or circRNA), and mRNA are positively correlated, while lncRNA (or circRNA), and miRNA are negatively correlated. We calculate the correlation between DEmiRNAs-DElncRNAs, DEmiRNAs-DEcircRNAs, and DEmiRNAs-DEmRNAs by Pearson correlation test (Pearson correlation coefficients < −0.8, *p* value < 0.05). Subsequently, the targeting relationship of DElncRNAs-DEmiRNAs, DEcircRNAs-DEmiRNAs, and DEmiRNAs-DEmRNAs was predicted by miRanda software, and the ceRNA pairs (*p* value < 0.05) were screened according to the ceRNA score analysis results [32, 33]. Then, we calculated the correlation between DElncRNA (or DEcircRNA) and mRNA using Pearson correlation test (Pearson correlation coefficient > 0.8, *p* value < 0.05), and the results intersected with the results obtained from ceRNA score screening. Using DElncRNAs, DEmiRNAs, and DEmRNAs screened according to the above threshold, we constructed the lncRNA-related ceRNA regulatory networks. Another circRNA-associated ceRNA network was composed of DEcircRNAs, DEmiRNAs, and DEmRNAs. These ceRNA networks of ceRNA pairs were constructed and visualized using Cytoscape (version 3.9.1, the Cytoscape Consortium, San Diego, USA).

### Protein–protein interaction (PPI) analysis and validation of the DEmRNAs in ceRNA networks

Based on the STRING database (https://string-db.org/), PPI analysis was conducted for DEmRNAs in all lncRNA-associated ceRNA networks, and all circRNA-associated ceRNA network. Based on the results of the STRING website, we imported the PPI results into Cytoscape construct network. Based on the centrality and importance of genes in the PPI network, we selected the CytoHubba plug-in to calculate the top ten hub genes using the MCC algorithm. Then we selected the DEmRNA of interest to carry out immunohistochemical verification in normal tissues and trauma tissues.

### Statistical analysis

Except for the qRT-PCR analysis described above, all statistical analyses were performed using R software (version 4.1.2, Boston, Massachusetts, USA). Continuous data were compared with an independent samples *t* test. *p* < 0.05 was considered to indicate a statistically significant difference.

## Results

### Construction of the rat TULP model

**Fig. 2:**
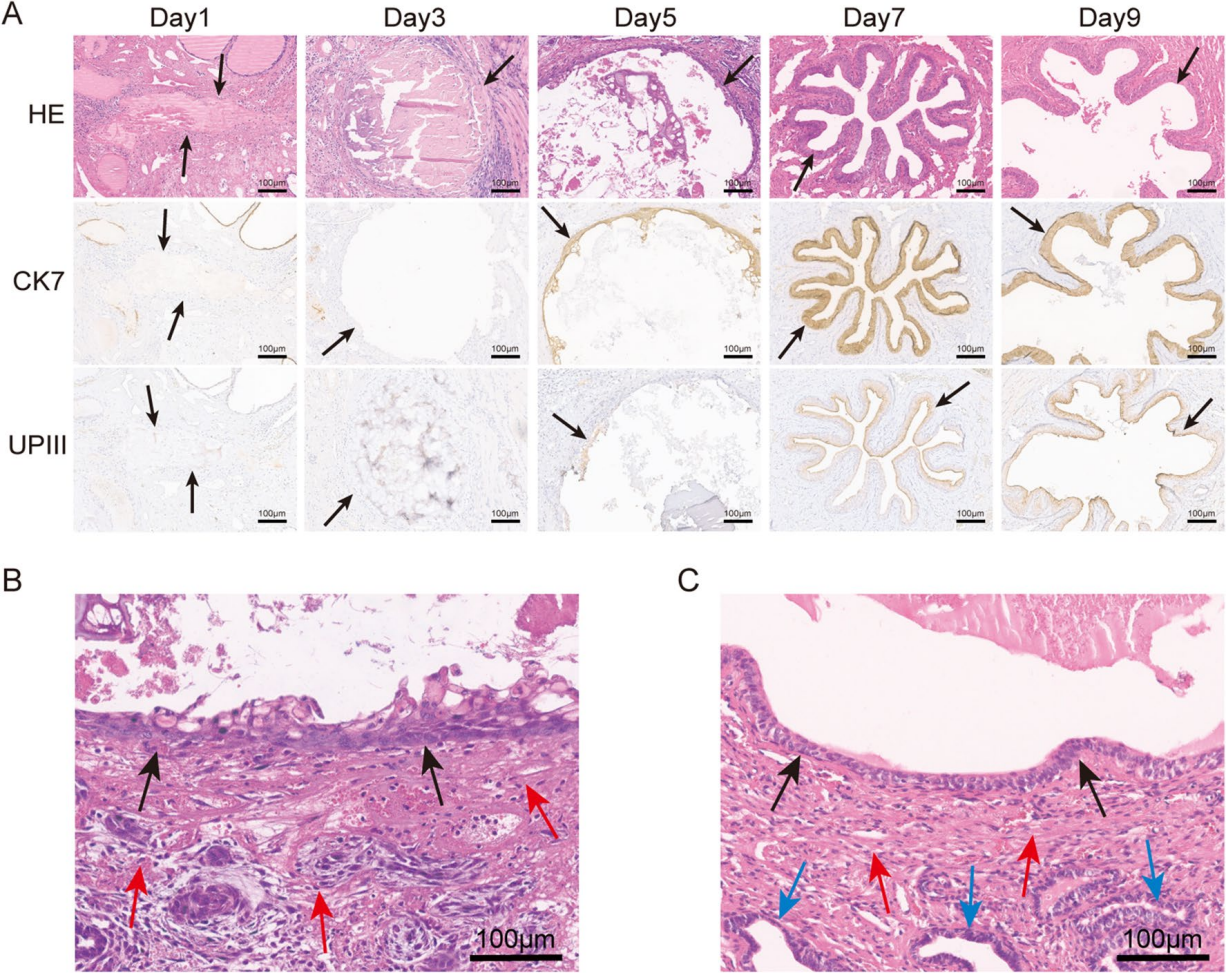
Pathological examination of wound tissues after prostatectomy in rats. **A** The prostatic urethra wound pathological changes were observed by HE staining and immunohistochemistry of CK7 and UPIII at 5 different time points; **B** Traumatic tissue of the prostatic urethra on the 5th postoperative day in the TULP group; **C** The normal tissue of the prostatic urethra on the 5th post-sham operation in the sham group. Black arrows: the trabecular surface or regenerating epithelium, red arrows: the stroma, blue arrows: the prostatic ducts

CK7 is a marker of lumen epithelium and also considered as an early marker of urothelium epithelial cells [34, 35]. The new cells express CK7 on the wound surface indicated that they were epithelial cells, and it can also be considered as early urothelium epithelial cells. UPIII is a marker of the intermediate or terminal stages of urothelium epithelial cells [35]. The new epithelial cells on the wound express UPIII indicated that they have become mature urothelium epithelial cells. As shown in Fig. 2A, HE staining showed that the stroma and duct structures in the wound demonstrated unclear demarcation and were replaced by substantial coagulative necrosis and inflammatory exudation on postoperative day 1, and original uroepithelial cells had disappeared. CK7 and UPIII staining confirmed the absence of epithelium on the wound.

HE staining suggested that no regenerated epithelial cells were present on the wound surface, and a large number of inflammatory cells were seen infiltrating the stroma closer to the wound surface on postoperative day 3, but coagulative necrosis was significantly reduced. Similarly, CK7 and UPIII staining confirmed that there was no regenerated epithelium on the wound surface.

HE indicated that the 1–3 layers of new epithelial cells lacked polarity on the wound surface on postoperative day 5, and a large number of inflammatory cells were still seen infiltrating the wound. However, the regenerated epithelial cells expressed CK7 and partially expressed UPIII.

HE revealed regenerated and thickened epithelial cells with polarity in the 4th–6th layers on postoperative day 7. The number of inflammatory cells in the stroma of the wound was significantly reduced, and a large number of dilated blood vessels were distributed around the wound surface. The regenerated epithelial cells expressed CK7 and UPIII.

HE showed that the regenerated epithelial cells were polarized with full cytoplasm, and the stroma and duct structure around the trauma were clear on postoperative day 9. The number of inflammatory cells was sparse, and the regenerated epithelium highly expressed CK7 and UPIII.

Compared to the sham group, the wound surface of the TULP group was covered with new epithelial cells on postoperative day 5, but the epithelial cells were not polarized, were disorganized, and the cytoplasm of the new epithelial cells was full, while the tissue structure of the trauma was blurred. Inflammatory cells infiltrated the stroma, and more new blood vessels were distributed in the wound with vasodilation and vascular congestion (Fig. 2B, C).

### Differential expression profiles of lncRNA, circRNA, miRNA, and mRNA and functional enrichment analysis of DEmRNA

**Fig. 3:**
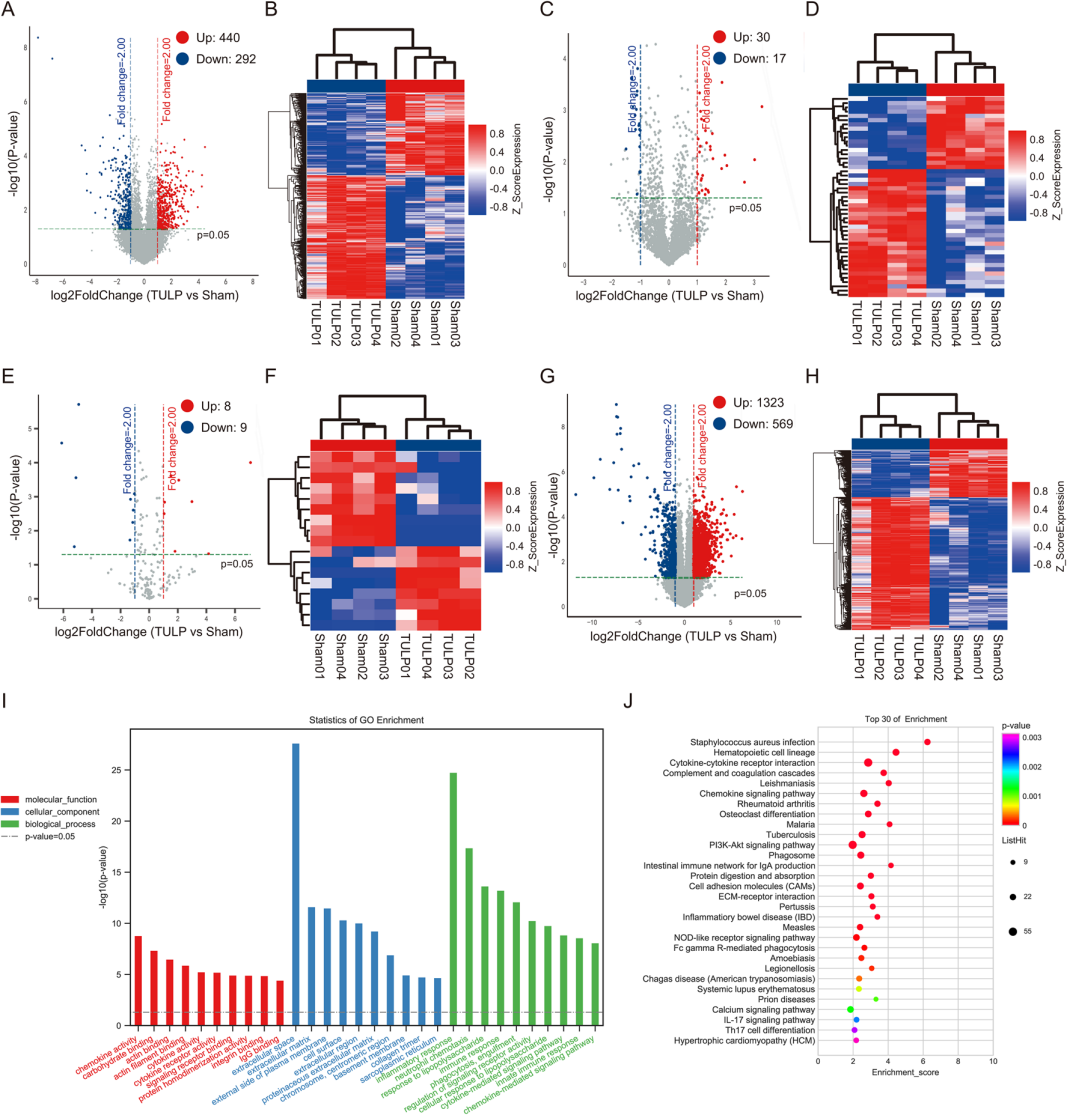
Identification and functional enrichment analyses of differentially expressed genes. Volcano map and heat map of differentially expressed lncRNAs (**A**, **B**), circRNAs (**C**, **D**), miRNAs (**E**, **F**), and mRNA (**G**, **H**). Volcano plot (**A**, **C**, **E**, **G**) show up-regulated (red arrow) and down-regulated (blue arrow) genes with the numbers, respectively. Columns clustering of the heatmap (**B**, **D**, **F**, **H**) indicated different group by colour (sham group = red, TULP group = blue), and the gene expression across rows in the heatmap are coloured according to the *z*-score. Red arrow: higher than the mean expression, blue arrow: lower than the mean expression, white arrow: the mean expression; **I** The top 10 significant terms of GO analysis (MF/CC/BP) of EDmRNAs; **J** The top 30 significant terms of KEGG pathway analysis of DEmRNAs. MF: molecular function, CC: cellular component, BP: biological process

DElncRNAs, DEcircRNAs, DEmiRNAs, and DEmRNAs were screened under the conditions of |FoldChange|> 2 and *p* value < 0.05. Overall, we identified 732 DElncRNAs (440 up-regulated and 292 down-regulated lncRNAs), 47 DEcircRNAs (30 up-regulated and 17 down-regulated circRNAs), 17 DEcircRNAs (8 up-regulated and 9 down-regulated miRNAs), and 1892 DEmRNAs (1323 up-regulated and 569 down-regulated mRNAs) in the TULP group compared with the Sham group (Fig. 3A–H), and the top 10 up- or down-regulated DERNAs (DElncRNAs, DEcircRNAs, DEmiRNAs, and DEmRNAs) are shown in Table S2.

We used GO and KEGG pathway analysis to analyze the EDmRNAs. GO analysis demonstrated that inflammatory response, neutrophil chemotaxis, response to lipopolysaccharide, immune response and extracellular space of DEmRNAs were the most significant GO terms (Fig. 3I). KEGG pathway enrichment analysis revealed that the most highly enriched pathways were cytokine–cytokine receptor interaction, chemokine signaling pathway, PI3K-Akt signaling pathway, ECM-receptor interaction and IL-17 signaling pathway (Fig. 3J).

### Quantitative real-time PCR (qRT–PCR) verification of differentially expressed lncRNAs, circRNAs, miRNAs and mRNAs

**Fig. 4:**
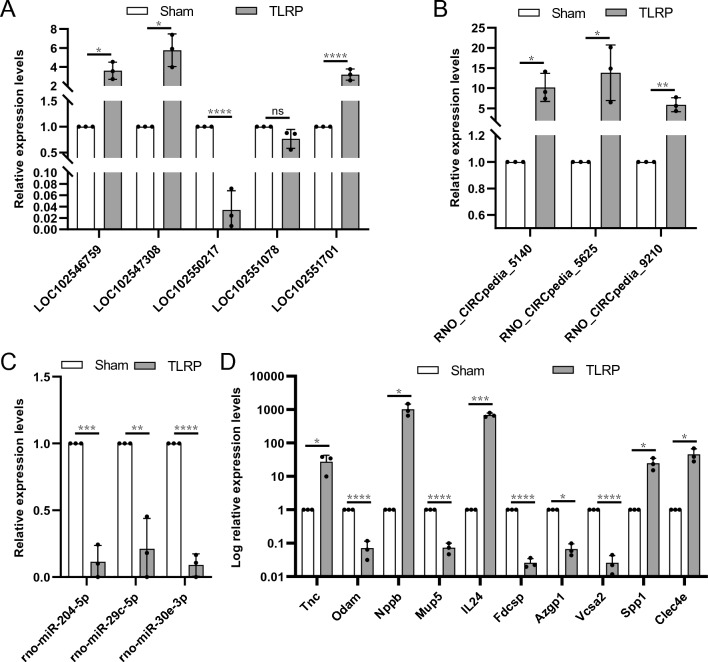
DERNAs were randomly selected for PCR validation. **A** DElncRNAs; **B** DEcircRNAs; **C **DEmiRNAs; **D** DEmRNAs, the *y*-axis was log10 transformed to enhance visualization (*p < 0.05, **p < 0.01, ***p < 0.001; ****p < 0.0001; ns, no significance)

In order to verify the reliability of microarray data results and based on the evaluation of the total number of differentially expressed genes in four different types, 5 DElncRNAs, 3 DEcircRNAs, 3 DEmiRNAs, and 10 DEmRNAs were randomly selected for qRT–PCR verification. As shown in Fig. 4, 3 lncRNAs, 3 circRNAs and 5 mRNAs were significantly up-regulated, and 1 lncRNA, 3 miRNAs and 5 mRNAs were significantly down-regulated versus the sham group, demonstrating the same trend as that seen in the microarray data. However, the difference in the LOC102551078 value was not statistically significant between the two groups.

### Construction and enrichment analysis of the DElncRNA (DEcircRNA)-DEmiRNA-DEmRNA ceRNA regulatory networks

**Fig. 5:**
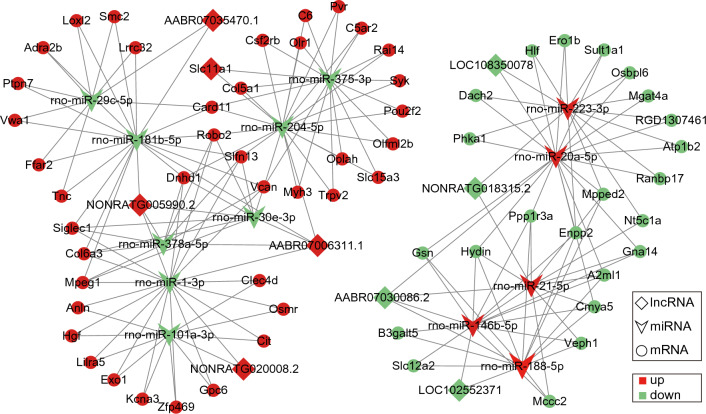
The lncRNA-miRNA-mRNA ceRNA regulatory network: rhombuse represents lncRNA, concave quadrilateral represents miRNA, and circle represents mRNA. Red indicates up- and green down-regulation, respectively

**Fig. 6:**
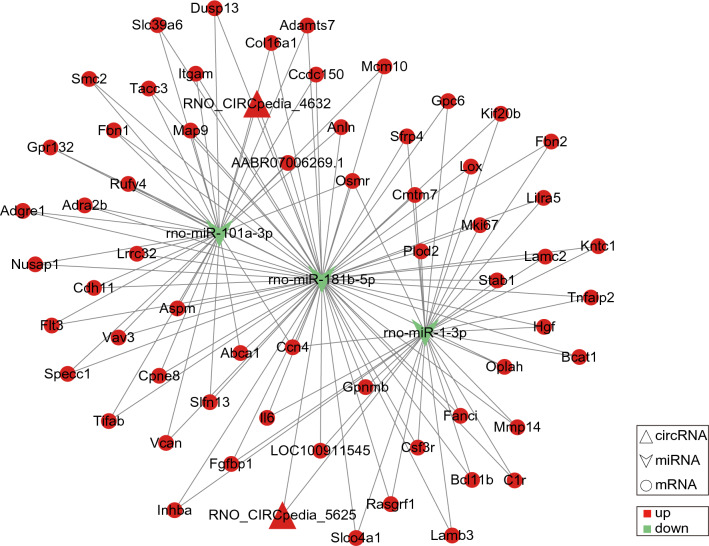
CeRNA interaction network of circRNA-miRNA-mRNA: triangle, concave quadrilateral, and circles denote circRNA, miRNA, and mRNA, respectively. Red and green colors denote up-regulated or down-regulated genes, respectively

CeRNA network construction was established based on the theory that “miRNA sponges” (lncRNAs or circRNAs) can sponge miRNAs to regulate specific target mRNAs. Based on the ceRNA hypothesis, we constructed ceRNA pairs from the whole transcriptome data, and calculate ceRNA score of an lncRNA-mRNA pair targeted by miRNAs (Table S3). For better visualization, we selected ceRNA pairs with top 100 ceRNA scores to construct and display parts of lncRNA-related ceRNA network, including 9 lncRNAs, 13 miRNAs, and 63 mRNA from DERNAs (Fig. 5). At the same time, based on the whole transcriptome data, we constructed circRNA-related ceRNA networks, including 2 circRNAs, 3 miRNAs and 60 mRNA from DERNAs (Fig. 6, Table S4). The scatter plots checking Pearson correlation for all significant pairs were shown in the Online Resource 1.

**Fig. 7:**
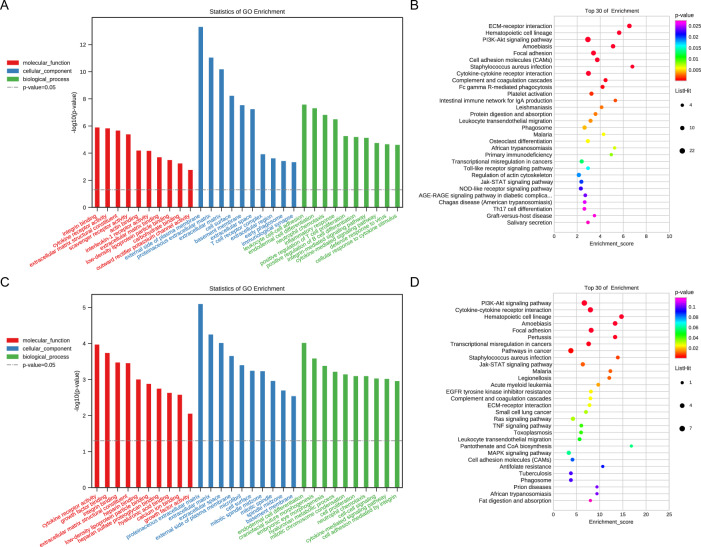
GO and KEGG pathway enrichment analysis mRNAs in ceRNA networks. **A**, **B** GO and KEGG analysis in the lncRNA-related ceRNA networks; **C**, **D** GO and KEGG analysis in the circRNA-related ceRNA networks

Furthermore, DEmRNAs from lncRNA-and circRNA-associated ceRNA networks were selected for GO and KEGG pathway enrichment analyses, respectively. The significantly enriched GO terms associated with lncRNA–miRNA-mRNA networks were leukocyte cell–cell adhesion, endodermal cell differentiation, neutrophil chemotaxis, inflammatory response and positive regulation of T-cell proliferation (Fig. 7A). Subsequently, KEGG pathways related to the mRNAs of LncRNA-miRNA–mRNA networks included the ECM-receptor interaction, hematopoietic cell lineage, PI3K-Akt signaling pathway, amoebiasis and focal adhesion (Fig. 7B). We carried out GO enrichment analysis of mRNAs in the circRNA-miRNA–mRNA network, and the results indicated that the DEmRNAs were enriched in endodermal cell differentiation, craniofacial suture morphogenesis, embryonic eye morphogenesis, hyaluronan metabolic process and mitotic chromosome condensation (Fig. 7C). Moreover, the coding genes from the circRNA-miRNA–mRNA network were significantly enriched in the PI3K-Akt signaling pathway, cytokine–cytokine receptor interaction, hematopoietic cell lineage, amoebiasis and focal adhesion (Fig. 7D).

### PPI and immunohistochemistry analysis

**Fig. 8:**
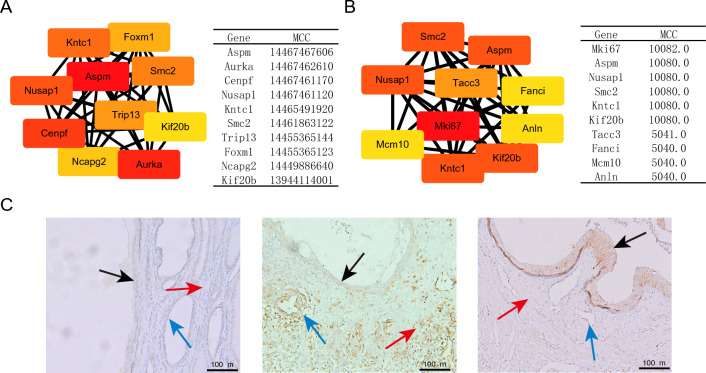
The top ten hub genes in the PPI network constructed from DEmRNAs in lncRNA-miRNA-mRNA network (**A**) and circRNA-miRNA-mRNA (**B**) were identified; **C** Immunohistochemistry of ASPM of wound tissues after prostatectomy in rats (left: normal, middle: 5 days, right: 9 days). Black arrows: urothelial cells, blue arrows: prostate ductal epithelial cells

According to the online STRING datavase (https://string-db.org/), the lncRNA–miRNA-mRNA networks-associated PPI network contained 1204 edges involving 347 nodes. Then, we used the Cytoscape plugin cytoHubba to identify 10 hub genes in the PPI network (Fig. 8A). The circRNA-miRNA-mRNA network-associated PPI network with 55 nodes and 77 edges was similarly established. The top 10 hub genes were identified from PPI network by CytoHubba (Fig. 8B). Interestingly, ASPM is the top 3 hub gene in both lncRNA- and circRNA-associated ceRNA networks-related PPI networks. ASPM is an important gene involved in the mitotic spindle [36]. ASPM is also considered to be the progenitor cell of urothelium [37]. The expression of ASPM in new epithelial cells means that its mitotic activity may increase, and it also suggests that new epithelial cells may have a close relationship with urinary tract epithelial progenitor cells. ASPM was validated using immunohistochemistry, and we found that ASPM was weakly expressed in the normal urothelial cells and prostate gland epithelial cells (Fig. 8C, left), but strongly expressed in the new prostate gland epithelial cells and stroma cells near the wound surface 5 days after surgery (Fig. 8C, middle), and this was consistent with the chip sequencing results. Interestingly, there was no significant expression of ASPM in the prostate gland epithelial cells and stroma cells of the wound 9 days after surgery, but ASPM was highly expressed in the newborn urothelial cells covering the wound surface (Fig. 8C, right).

## Discussion

Because BPH is a common disease of older men with high morbidity rates, the number of patients undergoing prostatectomy is large, and BPH is associated with substantial social and medical challenges. While surgery can resolve a patient's bladder outlet obstruction, complications can subsequently arise. With the advancement of technology, urinary incontinence, urethral perforation and other serious complications rarely occur. However, some complications cannot be significantly reduced with the advancement of technology or equipment, such as hematuria [8] and symptoms of urethral irritation [38]. Post-prostatectomy tissue repair reversed these complications in our previous study [39]. Although the prostate anatomy in rats is not identical to that in dogs and humans, the wound remaining after prostatectomy essentially represents resection of the uroepithelium and exposure of the prostatic ducts [40]. In the present study, we found that the early stages of repair mainly involved the removal of necrotic material from the wound and the recruitment of inflammatory cells to create a favorable repair microenvironment for wound repair, while there were no regenerating uroepithelial cells on the wound. At this point, the prostate duct near the trauma site is severely damaged, and the stroma contains many inflammatory cells. Regenerated epithelium appeared on the wound surface in the middle of the repair period, with high expression of CK7 but weak expression of uroepithelium-specific markers, suggesting a low probability of mature uroepithelial cells regenerating the uroepithelium in the prostatic duct. Our previous study revealed that regenerated uroepithelium after prostate trauma is more likely to originate from residual prostate tissue [39], and such cells with high expression of CK7 at the early stage of repair may be the important cells involved in nascent uroepithelium repair. By this stage of repair, the damaged prostatic ducts are largely repaired, because this repair process occurs rapidly. At the later stage of repair, the nascent uroepithelium is rapidly repaired and mature uroepithelial cells with high expression of the specific marker UPIII are present. Interstitial cells in the wound are also repairing rapidly, inducing the neovascularization and reconstruction of prostatic ducts. During the wound repair process, the cells in the wound cooperate with each other. For example, the new uroepithelium covering the wound reduces the stimulation of interstitial cells by urine, the neovascularization of abundant blood plasma facilitates the repair of uroepithelium and nonurethral epithelium, and the infiltration of inflammatory cells may participate in all the cellular repair processes. Overall, the animal model established in this study demonstrated the whole process of post-prostatectomy trauma repair, similar to our previous model established in dogs and mice [20, 41]. The repair time of prostate urethral wounds in rats and mice is similar, but the repair time of wounds in dogs is slower than that in rats and mice, which may be related to species. Our previous studies have primarily focused on TULP mouse models, but there are limitations in obtaining mouse wound tissue. Therefore, we constructed a rat TULP model for our research, which also provides a new perspective for TUPL-related research. This study established the first TULP model in rats, which will benefit further scientific research.

Past studies on the repair mechanism after prostatectomy have focused on the coding genes. For example, prostate epithelial cells and their high expression of CKIP-1 might reduce scar formation after prostate wound repair [20]. However, the overall study of the trauma repair microenvironment remains limited, and there is a lack of large-scale transcriptomic studies, complicating the study of molecular mechanisms in the prostate repair microenvironment and the search for new biomarkers to improve treatment. In the present study, we report transcriptome data involving lncRNAs, circRNAs, miRNAs and mRNAs after prostatectomy in rats for the first time. These differentially expressed RNAs might be closely associated with wound repair of after TULP. For example, Jimmy Lee et al. [42] suggested that lncRNA Pvt1 is involved in skin tissue homeostasis and wound repair. Takeshi Okada et al. [43] found that TNC promotes fibrosis and exerts reparative effects in an experimental aneurysm model via macrophage-induced migration and proliferation of smooth muscle cells. Chao Shi et al. [44] indicated that endothelial progenitor cell abdominal aortic aneurysm repair can be promoted by down-regulating miR-204-5p. Yun-Jie Shi et al. [45] found that IL6 can effectively improve the repair of intestinal epithelial injury. In addition, we validated the identified differential genes by qRT–PCR, confirming that the sequencing results are reliable. According to the GO enrichment analysis, DERNAs were mainly involved in Inflammation and immune response and were primarily located in the extracellular space and external side of the plasma membrane. In addition, based on the KEGG enrichment analysis, DERNAs were mostly enriched in Pathways related to cytokines and inflammation, which are involved in the repair process of post-prostatectomy wounds. Previous research identified that these pathways are related to tissue cell repair, such as repair of pulmonary artery endothelial cells [46], promoting cardiac repair postmyocardial infarction [47], and functional repair of spinal cord injury [48]. Interestingly, the pathways associated with prostate repair found in our previous studies in dogs were not significant in the present study, and we speculated that the differences might be due to species specificity as we used rats in the current study. Therefore, we believe that the translation from animal experiments to clinical applications requires further exploration and validation.

The ceRNA mechanism plays an important role in tissue regeneration and wound repair [49, 50]. However, up to now, there is no report on ceRNA research related to prostate wound repair. Our results showed that several ceRNA networks may be involved in the repair of the prostatic urethra after TULP, for example, in the ceRNA network with rno-miR-101a-3p as the core, the DElncRNAs (such as LOC102546759, and LOC108350774) and DEcircRNAs (RNO_CIRCpedia_4632) can regulate HGF through rno-miR-101a-3p. Hepatocyte growth factor (HGF) is a pleiotropic cytokine that has been extensively studied in wound repair. For instance, HGF can accelerated wound closure by promoting the growth of fibroblasts and promoted epithelial wound healing following mechanical corneal injury [51, 52]. Despite the above findings, more studies are needed to explore the role of ceRNAs mechanism in prostate wound repair.

In the lncRNA- and circRNA-associated ceRNA regulatory network, we found that the DEmRNAs in ceRNA network were associated with wound repair in the GO and KEGG analysis, such as leukocyte cell–cell adhesion, neutrophil chemotaxis, inflammatory response, Pl3K-Akt signaling pathway, and endodermal cell differentiation. Numerous studies have shown that almost all injuries, including minor injuries, cause an inflammatory response and immune infiltration, protecting tissue cells from microorganisms and activating regenerative signals for repair, the acute inflammation of the wound could usually promote the repair of the wound in an orderly manner [53–58]. Previous study proved that the PI3K-AKT pathway was a key factor in re-epithelialization, and played an important role in wound repair [59, 60]. Our previous studies have found that the urothelial epithelium of the prostate was completely loss after surgery, and the seed cells of re-epithelialization may be derived from certain types of cell in the prostate duct [39], while the human uroepithelium was differentiated from the endoderm [61], so the endodermal cell differentiation may be of great significance for re-epithelialization of prostatic urothelium after surgery.

ASPM,which encodes a centrosomal protein, plays a crucial role in the mitotic spindle during cell replication. As the hub gene of PPI network related to ceRNA network, ASPM may play an important role in the repair of prostate urothelial injury. Yan Li et al. [37] performed single-cell sequencing and found that ASPM-labeled basal cells might be the bladder uroepithelium progenitor cells. However, there is no report on ASPM in the repair of urinary tract epithelium in the prostate. In the past, we found that the new urinary tract epithelium after prostate trauma may come from some cells in the prostate duct, including prostate gland epithelial cells [40]. Interestingly, this study found that the expression of ASPM in the prostatic gland epithelial cells near the wound in the middle stage of repair was significantly higher than that in the normal prostatic gland epithelial cells, while the expression of ASPM in the neonatal urinary tract epithelial cells was significantly elevated in the late stage of repair, but there was no significant expression in other cells of the wound surface. In addition, the prostatic gland epithelial cells in the prostatic duct in the wound were close to the wound surface in anatomical position, therefore, we speculated that the prostate gland epithelial cells with high expression of ASPM may differentiate into urothelial cells and migrate to the wound surface to repair the adjacent urothelial cells after prostate trauma, but it is also possible that the repair of the prostate gland epithelial cells is earlier than the repair of the urothelial cells in the repair process, which is manifested by the high expression of ASPM in these two cells successively.

Molecular regulation, as a precise therapeutic strategy, can influence the development and progression of diseases by intervening in gene expression, regulating signaling pathways, or regulating intracellular metabolic processes [62–64]. These studies provide the foundation for developing treatment strategies targeting specific molecules and promote the implementation of personalized therapies. This study provides us with a deeper understanding of the molecular mechanisms of ceRNA related to prostate and urethral repair, which can be explored through the development of new drugs, gene therapy methods, and intervention strategies in the future. For example, by designing small molecule drugs or antibodies targeting specific molecules, we can selectively intervene in abnormal signaling pathways or inhibit pathological molecular interactions, thereby achieving precise treatment.

Despite the above results, our study has some limitations. First, the number of rats is limited because there were only 4 in each group, and a larger sample number is needed to validate our results. Second, different species might result in different results that must be verified in additional studies of other species. Third, this study used normal rats and further research is needed to explore the repair mechanism of the prostate urethra in rats under different hormone levels and prostate states in order to gain a more comprehensive understanding of the wound repair mechanism after prostatectomy. Finally, we did not discuss in depth the reason why the regeneration rate of the urinary tract epithelium of the rat prostate after trauma was faster than that of our previous studies on dogs [17].

## Conclusions

In this study, a novel rat model of TULP was constructed and the process of healing the prostatic urethral wound was observed. We constructed lncRNA- and circRNA-associated ceRNA networks in the rat TULP model. Additionally, we screened important target genes and pathways. These results may provide new animal models for wound repair after prostatectomy in the future, provide new insights for related molecular mechanism research, and help to provide new targets for clinical treatment.

### Supplementary information

Below is the link to the electronic supplementary material.

Supplementary file1 (RAR 59948 KB)

Supplementary file2 (DOCX 17 KB)

Supplementary file3 (XLSX 14 KB)

Supplementary file4 (XLSX 14 KB)

Supplementary file5 (XLSX 80 KB)

Supplementary file6 (XLSX 12 KB)

## Data Availability

Data from all microarray data are uploaded to the NCBI Gene Expression Omnibus (GEO) database (access number GSE209831). To review GEO accession GSE209831, go to https://www.ncbi.nlm.nih.gov/geo/query/acc.cgi?acc=GSE209831.
