# Supplementary material for: Construction of lncRNA- and circRNA-associated ceRNA networks in the prostatic urethra of rats after simulating transurethral laser prostatectomy (TULP)
Source: Mol Cell Biochem. 2023 Jul 6;479(6):1363–77. doi: 10.1007/s11010-023-04804-1 (PMC11224087; doi:10.1007/s11010-023-04804-1)
Supplement: Supplementary file 2 — Supplementary file2 (DOCX 17 KB) [file 11010_2023_4804_MOESM2_ESM.docx]

**READ ME**

This word you are currently reviewing, provides a list of all supplementary files to the manuscript.

The following data are supplementary materials for the manuscript to be submitted in ***Molecular and Cellular Biochemistry***. The title of the manuscript is ***Construction of lncRNA- and circRNA-associated ceRNA networks in the prostatic urethra of rats after simulating transurethral laser prostatectomy (TULP)*** *(Submission ID 9dd0de8e-0c31-415b-b3ab-8e406498e944)*.

The authors are XiaoHu Tang, ZhiYan Liu, Hao Liu, Heng Zhang, Ye Tian, ShuJie Xia, ZhaoLin Sun, GuangHeng Luo

****Corresponding author:***

*Guangheng Luo, Department of Urology Surgery, Guizhou Province People’s Hospital, Guiyang 550002, Guizhou Province, China; Email:* [*luoguangheng1975@126.com*](mailto:luoguangheng1975@126.com)

**Supplementary material Legends**

**Supplementary file 1: Table S1 (XLSX 15 KB)**

Primer sequences for quantitative real-time PCR

**Supplementary file 2: Table S2 (XLSX 15 KB)**

Top ten upregulated and downregulated differentially expressed lncRNAs, circRNAs, miRNAs, and mRNAs

**Supplementary file 3: Table S3 (XLSX 80 KB)**

LncRNA associated ceRNA pairs and ceRNA score

**Supplementary file 4: Table S3 (XLSX 13 KB)**

Table S4: circRNA associated ceRNA pairs and ceRNA score

**Supplementary file 5: Online Resource 1 (RAR 59,949 KB)**

The scatter plots checking Pearson correlation for all significant pairs from all ceRNA networks
